# Tribological and Electrochemical Characterization of UHMWPE Hybrid Nanocomposite Coating for Biomedical Applications

**DOI:** 10.3390/ma12223665

**Published:** 2019-11-07

**Authors:** Zahid Ahmed Baduruthamal, Abdul Samad Mohammed, A. Madhan Kumar, Mohamed A. Hussein, Naser Al-Aqeeli

**Affiliations:** 1 Department of Mechanical Engineering, King Fahd University of Petroleum & Minerals, Dhahran 31261, Saudi Arabia; zuwais@kfupm.edu.sa (Z.A.B.); naqeeli@kfupm.edu.sa (N.A.-A.); 2 Center of Research Excellence in Corrosion, Research Institute, King Fahd University of Petroleum & Minerals, Dhahran 31261, Saudi Arabia; madhankumar@kfupm.edu.sa (A.M.K.); mahussein@kfupm.edu.sa (M.A.H.)

**Keywords:** titanium, polymer, hybrid nanocomposite coating, tribology, UHMWPE

## Abstract

A new approach of using a polymer hybrid nanocomposite coating to modify the surface of titanium and its alloys is explored in this study. Electrostatic spray coating process is used to deposit the coating on the plasma-treated substrates for better adhesion. Ultra-high molecular weight polyethylene (UHMWPE) has been selected as the parent matrix for the coating due to its biocompatibility and excellent tribological properties. However, to improve its load-bearing capacity carbon nanotubes (CNT’s) (0.5, 1.5, and 3 wt.%) are used as reinforcement and to further enhance its performance, different weight percent of hydroxyapatite (HA) (0.5, 1.5, 3, and 5 wt.%) are introduced to form a hybrid nanocomposite coating. The dispersion of CNT’s and HA was evaluated by Raman spectroscopy and scanning electron microscopy. The electrochemical corrosion behavior of the nanocomposite coatings was evaluated by performing potentiodynamic polarization and electrochemical impedance spectroscopic tests in simulated body fluid. Tribological performance of the developed hybrid nanocomposite coating was evaluated using a 6.3 mm diameter stainless steel (440C) ball as the counterface in a ball-on-disk configuration. Tests were carried out at different normal loads (7 N, 9 N, 12 N, and 15 N) and a constant sliding velocity of 0.1 m/s. The developed hybrid nanocomposite coating showed excellent mechanical properties in terms of high hardness, improved scratch resistance, and excellent wear and corrosion resistance compared to the pristine UHMWPE coatings.

## 1. Introduction

Titanium and its alloys are used in several biomedical implants which replace damaged hard tissue since the 1970s. Pacemakers, bone plates, artificial/hip/knee joints, screws for fracture fixation, cardiac valve prostheses, dental and orthopedic implants, etc., are few examples from the field of biomedicine where titanium is being used extensively [1]. Titanium alloys are a better choice for orthopedic materials compared to stainless steel and cobalt alloys due to their excellent biocompatibility, corrosion resistance, and low modulus. Thus several components are manufactured from titanium alloys for biomedical application. However, poor tribological properties, lack of mechanical stability in the oxide layer and low abrasive resistance are some of the limiting factors associated with titanium alloys [2].

These drawbacks of titanium alloys may jeopardize the long-term use of the implant that could lead to medical complications, such as osteolysis and aseptic loosening, and may not satisfy clinical requirements. Thus, various surface modification techniques have been studied for titanium and its alloys [2].

The use of polymer coatings in engineering applications has significantly increased in the recent past, due to their remarkable properties such as excellent corrosion and wear resistance, low cost, self-lubricating properties, and the ability to coat onto any complex shapes [3]. However, to be used in biomedical applications, the polymer coatings should not only have good mechanical and tribological properties but should also be biocompatible. One of such polymers having a blend of both the above criteria is ultra-high molecular weight polyethylene (UHMWPE).

UHMWPE is used in several biomedical applications due to its high strength and bio-compatibility. In addition, its high stiffness and strength make it useful in structural materials and fiber production [4]. Its outstanding properties, such as abrasive resistance, notched impact strength, and low coefficient of friction [5], make it feasible to be used in highly stressed parts, for instance, in total joint replacement, UHMWPE is still commercially used in the manufacturing of cups and tibial inserts [6]. Despite its excellent tribological properties, the use of UHMWPE in biomedical applications has been hindered due to its low load-bearing capacity which causes the generation of wear debris particles leading to complications such as osteolytic lesions and radiographic loosening [7], thus tackling this issue has been the focus of considerable scientific research. Reinforcing carbon nanotubes (CNT’s) into the UHMWPE polymer matrix is one possible solution to overcome this issue [8]. Several studies have shown that the reinforcement of CNT’s into polymer matrices in bulk form [9] and in the coating form [10] has resulted in a significant improvement in the mechanical properties in terms of hardness and wear resistance, owing to the exceptional mechanical properties of CNT’s such as high tensile strength, high stiffness, excellent electrical and thermal properties [11].

Moreover to further improve the biocompatibility of the UHMWPE, researchers have added various additives, such as graphene oxide [12], graphene nano-platelet [13], zirconium particles [14], etc. Hydroxyapatite (Ca_10_(PO_4_)_6_(OH)_2_, HA) is one such astounding biomaterial due to its similar chemical semblance with bone and teeth. The biocompatibility and bioactivity of HA flourish osteoblasts, therefore, HA coatings have been used in several biomedical applications, such as dental implants, skeletal implants, bone repair scaffolds, body insert materials, etc. [15]. Lately, substantial research has been devoted to the development of HA-reinforced coatings such as magnesium oxide/HA [16], titania/HA [17], strontium/HA [18], polycaprolactone/HA [19] coatings, nanodiamond/HA [20], etc, and deposited on titanium and other biomaterials, where the mechanical properties are ensured by the metal substrates while HA contributes to enhancing the biocompatibility [21].

However, not much research has been conducted on exploring the feasibility of using the above two individual reinforcements together to get the synergy effect of both the reinforcements collectively. Hence, the objective of the present study is to assess the feasibility of using a hybrid nanocomposite coating on pure titanium and Ti6Al4V (commercially available) and a newly developed titanium alloy (Ti20Nb13Zr) [22] to enhance their tribological properties. UHMWPE has been chosen as the parent polymer matrix for the coating due to its biocompatibility and good tribological properties, thus the initial phase of this study will include characterization and tribological optimization of pristine UHMWPE using different loading conditions. In the second phase, single-walled CNT’s (0.5, 1.5 and 3 wt.%) are introduced into the UHMWPE matrix to enhance the load-bearing capacity of UHMWPE. This nanocomposite coating will then be optimized by tribological analysis for different compositions of CNT’s. The final phase of this study is to introduce 0.5, 1.5, 3, and 5 wt.% of HA and investigate the tribological performance of the prepared hybrid nanocomposites. It is to be noted that several researchers as mentioned above have found that the addition of HA helps in the improvement of biocompatibility of the parent matrix which is also expected in this study. However, no experiments were conducted in evaluating the biocompatibility of the developed hybrid nanocomposites in this study as the main objective of the present research is to evaluate the effect of the addition of CNT’s and HA on the tribological performance of the developed hybrid nanocomposites. The dispersion of the constituents is evaluated by Raman spectroscopy and SEM images, and the coatings are further characterized by hardness and scratch tests.

## 2. Experimental Methodology

### 2.1. Materials

Commercially available titanium sheets of titanium grade 2—ASTM F67 (pure titanium) and titanium grade 5—ASTM F136 (Ti6Al4V) with the dimensions of 1 m × 0.5 m × 0.003 m were purchased from Xi’an Saite Metal Materials Development Co. Ltd. (Xi’an, China). Gelatin machine was used to cut the samples into small 25 mm × 25 mm square samples. The third substrate used in this study was a newly developed and patented nanostructured titanium alloy (Ti20Nb13Zr) [22,23]. All the substrates were ground to an average surface roughness of (Ra) = 0.51 ± 0.04 µm

UHMWPE powder was purchased from the Good Fellow Corporation, Huntingdon, UK. The average particle size ranged between 80 and 90 µm. Carbon nanotubes (CNT’s) were purchased from Nanostructured and Amorphous Materials Inc., Houston, TX, USA. The diameter of the CNT’s ranged from 40 to 60 nm with a length of 1–2 µm and a specific surface area of 60–70 m^2^/g.

Hydroxyapatite (HA) was used as a second filler along with CNT’s, for enhanced mechanical properties of the hybrid coating. HA was prepared in-house using the method described by Adrian et al. [24]: 0.555 g of CaCl_2_ (Calcium chloride), 0.150 g of NaH_2_PO_4_ (Sodium dihydrogen phosphate) and 0.073 g of NaHCO_3_ (Sodium bicarbonate) were dissolved in 500 mL of distilled water. The solution was stirred for 24 h at 37 °C and 80 rpm. The precipitate was washed with deionized water at the end of the procedure and dried in an oven at 110 °C for 2 h. The quantity of HA yielded was very little, insufficient for this study, therefore, the constituents used for the preparation of HA were multiplied by ×50, which resulted in a higher yielding of HA. The characteristic appearance of the powder was white in color and had a plate-like structure with a thickness ranging from 0.3 to 0.5 µm.

### 2.2. Hybrid Nanocomposite Powder Preparation

For the preparation of nanocomposite powders, 10 g of UHMWPE was reinforced with X wt.% of CNT’s. X g of CNT’s was weighed and emptied into a beaker containing 50 mL of ethanol and sonicated for 10 min using a probe sonicator. After the sonication process was completed, the solution was magnetically stirred at 1000 rpm and the required quantity of UHMWPE powder was added gradually, and the magnetic stirring process was continued for 60 min followed by a post-heat treatment process to completely evaporate the ethanol, leaving the UHMWPE reinforced with CNT’s nanocomposite powder which was collected and stored for subsequent characterization and tests.

For the preparation of the hybrid nanocomposite powders, 10 g of UHMWPE reinforced with X wt.% CNT’s and Y wt.% HA the method used by Kwok. et al. [25] was employed. Y grams of HA was added to 50 mL of ethanol and stirred using a magnetic stirrer for 10 min. After the stirring process, the solution of ethanol and HA was sonicated for 30 min. X grams of CNT’s was then added to the above solution containing ethanol and HA and sonicated for another 30 min. After the sonication process, the solution was subjected to magnetic stirring followed by the post heat treatment process to evaporate ethanol and obtain the hybrid nanocomposite powders. For the ease of understanding, codes were assigned to each of the compositions used in this study as shown in Table 1.

### 2.3. Coating Procedure

“Craftsman” electrostatic powder spray coating gun (Model no 17288) (Craftsman industrial Miami, FL, USA). was used to coat the samples. Prior to coating, all the samples were ultrasonically cleaned, dried and subjected to air-plasma treatment (Harrick Plasma, Ithaca, NY, USA) for 15 min at an RF power of 30 W and pre-heated for 5 min at 180 °C for better adhesion of the coating. After the spraying of the powder with a specific composition, the samples were cured at 180 °C for 30–35 min followed by air cooling to further characterize the final specimens [26].

### 2.4. Thickness Measurements

The thickness of the coating was determined using a field emission scanning electron microscope (FE-SEM) (Oxford Instruments, Oxfordshire, UK) and confirmed by the dry film thickness gauge Elcometer 456 (Electrometer Ltd., Manchester, UK).

Two samples of each composition were used and three measurements were recorded for each sample. The average value of the thickness is reported. Pristine UHMWPE had an average coating thickness of 142 ± 4 µm whereas, the nanocomposite coating and the hybrid nanocomposite coating had an average coating thickness of 181 ± 4 µm and 185 ± 4 µm, respectively.

### 2.5. Tribological Characterization

Bruker UMT-3 Tribometer (Bellerica, MA, USA), with a ball on disk arrangement, was used for the tribological tests. A 440C stainless steel ball with a 6.3 mm diameter and RC62 hardness was used as a counter-face. The coatings were tested under different loads of 7, 9, 12, and 15 N respectively to optimize the loadings of the reinforcements. 

The criteria used in this study to evaluate the coating failure include a sudden spike in the friction coefficient, suggesting a metal to metal contact or too many fluctuations in the coefficient of friction (COF) graph. To ascertain the coating failure optical microscopic assessment for wear and tear on the counterface ball, wear track analysis coupled with energy dispersive X-ray (EDX) analysis are used. Wear tests were performed on three samples of each composition, and the average value of the coefficient of friction and specific wear rate are reported.

Wear depth, wear volume, and specific wear rates were calculated using 3D optical profilometer (GTK-A, Bruker, Bellerica, MA, USA). Specific wear rates are calculated by initially finding the area under the curve for a 2D plot which is provided by the computer and multiplying it with the track circumference, i.e., 2πr, where r stands for the track radius, to calculate the wear volume. The wear volume is then divided by the applied normal load and distance traveled by the ball as shown in the equation below:

(1) $$Specific\;Wear\;rate=\;\frac{Wear\;volume}{Applied\;Normal\;load\;\times Distance\;}\;=\;\frac{mm^{3}}{Nm}\;$$

Raman spectroscopy (Thermo scientific DXR 455 nm, Waltham, MA, USA) was performed for UHMWPE reinforced with CNT’s coatings to investigate the interaction of CNT’s with the UHMWPE matrix and scanning electron microscope (Tescan VEGA3, Brno, Czech Republic) was used to analyze the dispersion of CNT’s and HA in the UHMWPE Matrix. Prior to SEM imaging, the samples were sputter-coated with gold using the “Fine Coat Ion Sputter JFC-1100”.

### 2.6. Hardness Measurement

Vickers hardness tests were conducted on the coated sample using the Micro-Combi Tester (CSM instruments, Portland, OR, USA) with a contact force of 0.01 N and an approach speed of 16.6 µm /min. The maximum applied load was 0.1 N with a loading and unloading rate of 0.20 N/min. The measurement was carried out on three samples of each type with the average value of 20 reading at different locations.

### 2.7. Scratch Test

Linear, progressive scratch test was performed for the optimized coatings using the Micro-Combi to ascertain the adhesion of the coatings. A rigidly mounted diamond having a Rockwell C geometry with a radius of 100 µm was used as the indenter to perform these tests. An initial load of 0.03 N and an end load of 30 N with a loading rate of 15 N/m and a scratch length of 10 mm were defined as test parameters.

### 2.8. Electrochemical Corrosion Analyses

Electrochemical corrosion analyses were carried out using a typical three-electrode cell with the potentiostat/galvanostat/ZRA (Reference 3000) (Gamry Instruments Philadelphia, PA, USA). As a reference electrode, a saturated calomel electrode (SCE) was used, whereas a graphite rod was used as a counter electrode. For the purpose of electrochemical characterization, the simulated body fluid (SBF) was used as an electrolyte. Using previous reports, the preparation of SBF and the procedure for performing the experiments were adopted. Each specimen had an exposed area of 1.76 cm^2^. Monitoring of the open circuit power (OCP) was conducted for about 30 min. A frequency range from 100 kHz to 10 mHz was used to evaluate the electrochemical impedance spectroscopy (EIS) with a 10 mV amplitude sinusoidal AC voltage. In order to verify reproducibility, electrochemical corrosion tests were repeated at least three times.

## 3. Results and Discussion

### 3.1. Evaluation of Interaction of CNT’s with UHMWPE Polymer Matrix Using Raman Spectroscopy

**Figure 1:**
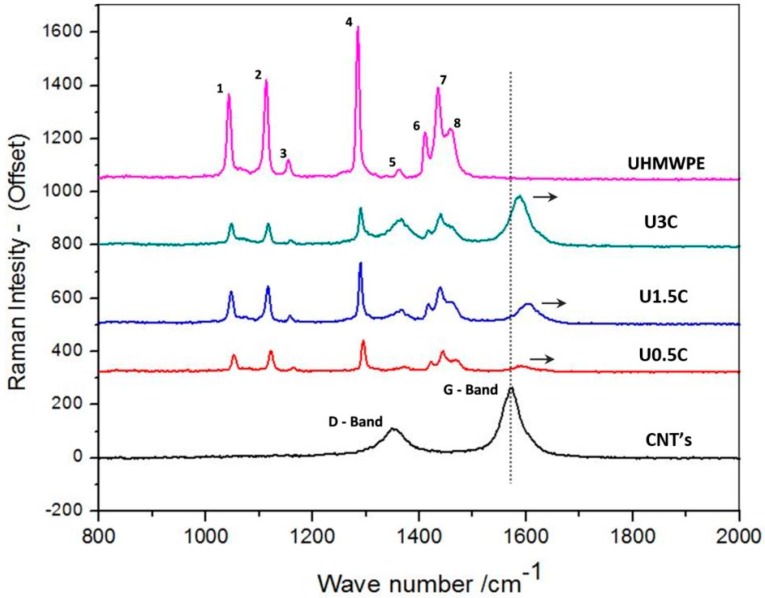
Raman spectroscopy peaks of pristine ultra-high molecular weight polyethylene (UHMWPE), UHMWPE reinforced with 0.5, 1.5, and 3 wt.% capacity carbon nanotubes (CNT’s).

Raman spectroscopy was used to study the interfacial interaction between the UHMWPE matrix and CNT’s. The characteristic Raman spectrum for the pristine UHMWPE and UHMWPE with the different loadings (0.5, 1.5, and 3 wt.%) of CNT’s are shown in Figure 1. As observed from the spectrum of pristine UHMWPE, points 1 and 2 are associated with the asymmetric and symmetric stretching modes of the C–C bond, while points 3 to 8 are subsequent to the twisting and bending modes of CH_2_. 

The Raman spectrum of only CNT’s displayed two characteristic peaks. The first peek at 1357 cm^−1^ designated as the D-band determining disordered graphite structures, the second peak centered at 1574 cm^−1^ assigned the G-band is correlated with the tangential C–C bond stretching motions [27].

In addition to 1.5 wt.% CNT’s, a maximum shift of 27 cm^−1^ in the position of the G-band peak is observed. The shifting of the G-Band peak to a higher frequency can be attributed to the disentanglement and extrication of CNT’s as a result of successive dispersal in the UHMWPE matrix. The up-shift of the G-band can also represent stronger compressive forces associated with the UHMWPE chains on CNT’s, indicating the intercalation of the polymer into nanotube bundles [28]. However, for 3 and 0.5 wt.% CNT’s, the upshift of the G-band was only 19 cm^−1^ and 14 cm^−1^ respectively, which is comparatively a low shift than 1.5 wt.% CNT’s, suggesting less interaction of the CNT’s with the UHMWPE matrix for the 3 and 0.5 wt.% CNT’s.

### 3.2. Dispersion Analysis of CNT’s in UHMWPE Matrix Using SEM

**Figure 2:**
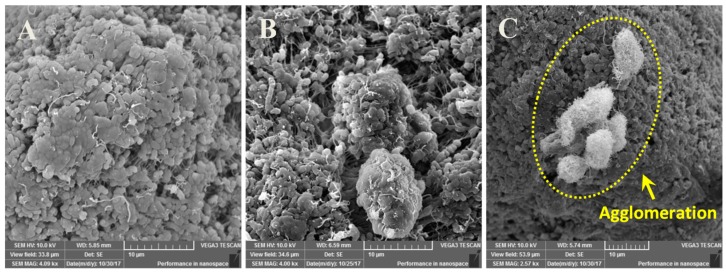
SEM images showing the dispersion of (**A**) 0.5 wt.%, (**B**) 1.5 wt.% and (**C**) 3 wt.% of CNT’s in UHMWPE polymer matrix.

SEM analysis of the nanocomposite powders of UHMWPE reinforced with different loadings (0.5, 1.5, and 3 wt.%) of CNT’s was conducted to ascertain the dispersal of CNT’s in the UHMWPE polymer matrix. The SEM images are shown in Figure 2. It can be observed from Figure 2A that for the U0.5C nanocomposite powders, the CNT’s seem to be evenly distributed without any apparent agglomerations, which is also observed for the U1.5C nanocomposite powders, as can be seen from Figure 2B. The presence of individual CNT’s in different locations attributes to good desperation and almost negligible agglomeration, suggesting that the sonication process employed is effective in dispersing the 0.5 wt.% and 1.5 wt.% CNT’s in the UHMWPE polymer matrix. However, when the loading of CNT’s was increased to 3 wt.% traces of agglomeration were observed, as shown in Figure 2C.

### 3.3. Tribological Characterization of Pristine UHMWPE Coating

**Figure 3:**
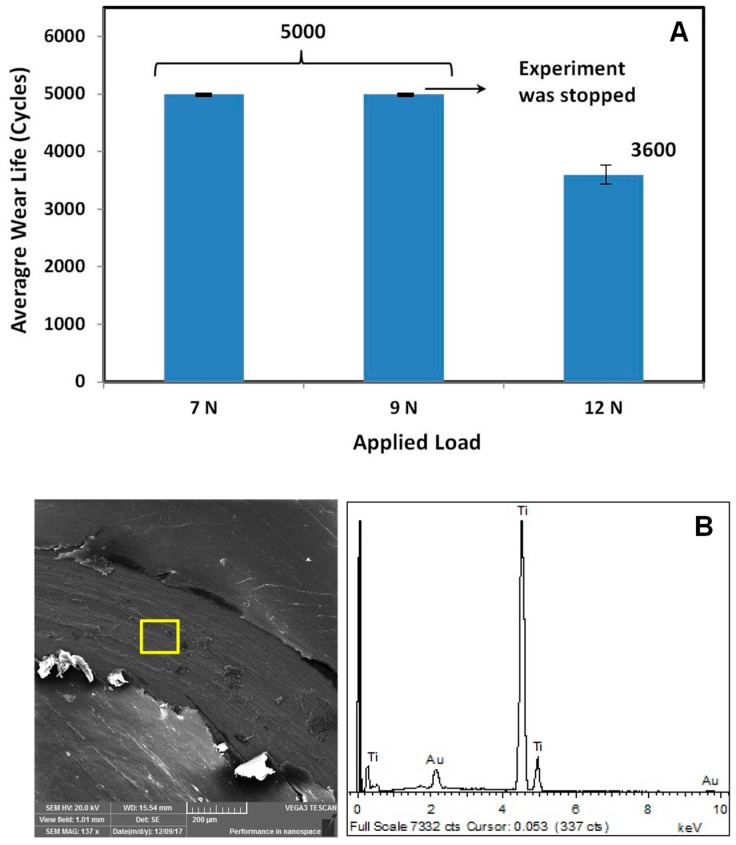
(**A**) Average wear life as a function of normal load, (**B**) SEM image of the wear track and energy dispersive X-ray (EDX) spectrum of pristine UHMWPE coating after a wear test at a normal load of 12 N and a sliding velocity of 0.1 m/s.

Initially, wear tests were performed at different normal loads of 7, 9, and 12 N, respectively, to determine the load-bearing capacity of the pristine UHMWPE coating. Three samples were tested for each loading condition. Tests were conducted at a constant sliding velocity of 0.1 m/s for 5000 cycles. Figure 3A shows the average wear life as a function of the applied load for pristine UHMWPE. It can be observed that the pristine UHMWPE coating did not fail for 5000 cycles at a load of 7 and 9 N respectively. The test was stopped after 5000 cycles for 7 N and 9 N loads in view of the nano-failure of the coating. However, increasing the normal load to 12N the pristine UHMWPE coating failed after ~3600 cycles, which was confirmed by EDX analysis conducted on the wear track. Figure 3B shows a clear peak of titanium, the material of the substrate indicating the failure of the coating, which can be ascribed to a combination of adhesive and abrasive wear resulting in the peeling off and plowing of the coating.

### 3.4. Tribological Characterization of UHMWPE/CNT’s Nanocomposite Coating

To further enhance the load-bearing capacity of the pristine UHMWPE coating and enhance its tribological performance, different loadings of CNT’s (0.5, 1.5, and 3 wt.%) were introduced into the UHMWPE matrix and three samples for each composition were tested at a load of 12 N initially with a sliding velocity of 0.1 m/s for 50,000 cycles.

**Figure 4:**
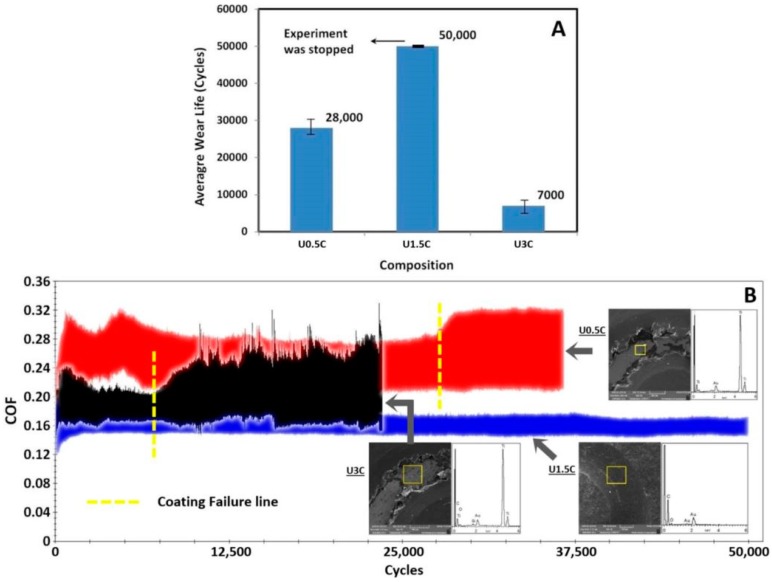
(**A**) Typical coefficient of friction (COF) graphs, (**B**) SEM images of wear track, EDX analysis, and average wear life for nanocomposite coatings recorded after a wear test conducted at a normal load of 12 N and a sliding velocity of 0.1 m/s for 50,000 cycles.

**Figure 5:**
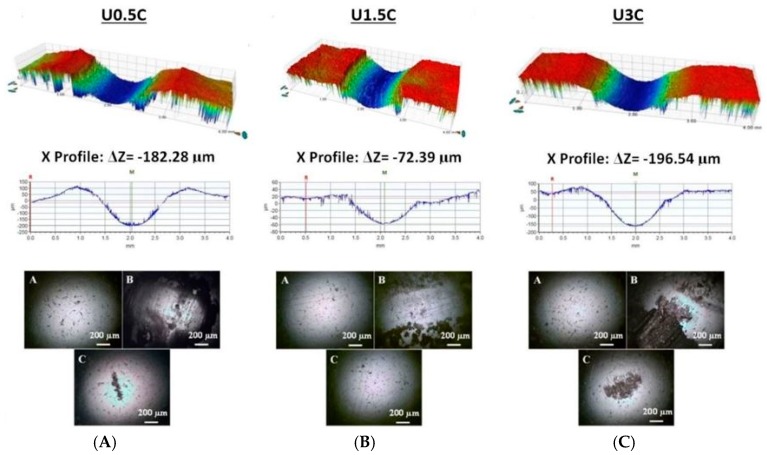
3D and 2D profiles and counterface ball images after the wear test conducted on UHMWPE nanocomposite coating, recorded at a normal load of 12 N and a sliding velocity of 0.1 m/s for 50,000 cycles. (**A**) U0.5C; (**B**) U1.5C; (**C**) U3C.

It was observed that an addition of 0.5 wt.% CNT’s into the UHMWPE polymer matrix was able to improve the wear life of the coatings as compared to the pristine UHMWPE coatings. However, the U0.5C coatings failed at ~28,000 cycles. A combination of the adhesive and abrasive mode of failure of the coating can be observed in the SEM image as shown in Figure 4B, and the EDX analysis confirms the exposure of the substrate. Optical profilometry was also conducted on the wear tracks after the wear tests and the 3D and 2D wear profiles for the different nanocomposite coatings are shown in Figure 5. The failure of the U0.5C nanocomposite coating is also confirmed by the 2D profile, which shows a profile depth of ~182 µm (Figure 5) which is approximately the coating thickness. The optical images of the counterface ball sliding against the coating recorded before the test and after the test (before cleaning and after cleaning) for a typical run are shown in Figure 5A–C. A good amount of material pullout is clearly visible on the counterface ball (Figure 5B) and a scar mark indicating a metal to metal contact can also be seen as shown in Figure 5C. The failure of the UHMWPE nanocomposite coating reinforced with 0.5 wt.% CNT’s can be attributed to the less amount of CNT’s in the UHMWPE polymer matrix, resulting in an inefficient anchoring of the polymer chains leading to more material pull out and ultimately resulting in the failure of the coating.

However, when the loading of CNT’s was increased to 1.5 wt.% the nanocomposite coating did not fail even until 50,000 cycles as can be seen from Figure 4B. The non-failure of the coating can also be confirmed from the EDX spectrum conducted on the wear track as shown in Figure 4B and also from the 2D profile of the wear track which shows a wear track depth of ~72 µm which is much lower than the coating thickness. The optical images of the counterface ball do not show any scar mark suggesting no metal to metal contact between the ball and the substrate. This improvement in the wear resistance of the nanocomposite coating can be attributed to the uniform distribution of the CNT’s in the UHMWPE polymer matrix resulting in a good interaction between the matrix and the CNT’s as confirmed earlier by the SEM and Raman spectroscopy analysis. 

However, on further increasing the loading of CNT’s to 3 wt.% the nanocomposite coating failed early, only after ~7000 cycles. The failure of the coating can also be confirmed from the EDX spectrum conducted on the wear track as shown in Figure 4B and also from the 2D profile of the wear track which shows a wear track depth of ~196 µm which is greater than the coating thickness. The optical images of the counterface ball also show a scar mark suggesting a metal to metal contact. This sudden deterioration in the tribological performance of the nanocomposite coating with an increase in the loading of CNT’s to 3 wt.% can be attributed to the agglomerations of CNTs, in the UHMWPE matrix confirmed from the SEM and Raman spectroscopy results discussed earlier. Agglomerated CNT’s in the polymer matrix create a hard and a soft phase in the coating, resulting in an uneven morphology which consequently results in the coating failure. 

**Figure 6:**
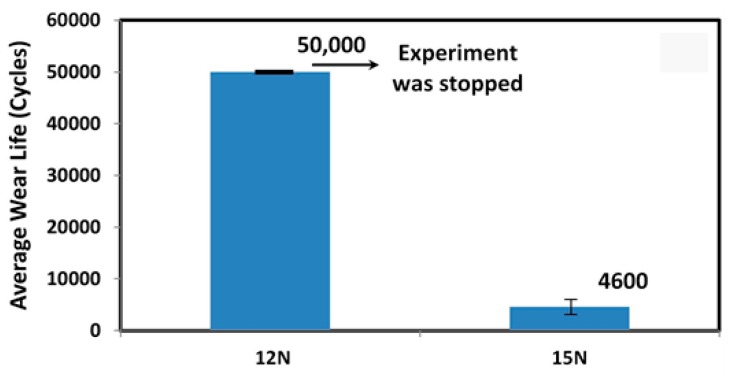
Average wear life of U1.5C coating recorded after a wear test conducted at a normal load of 15 N and a sliding velocity of 0.1 m/s for 50,000 cycles.

From the above wear tests conducted on the UHMWPE nanocomposite coating loaded with different amounts (0.5, 1.5, and 3 wt.%) of CNT’s, it was concluded that a loading of 1.5 wt.% of CNT’s showed the best tribological performance at a normal load of 12 N and a sliding velocity of 0.1m/s. However, to further evaluate the U1.5C nanocomposite coating at higher loads, wear tests were conducted at a normal load of 15 N and a sliding velocity of 0.1m/s. Figure 6 shows the average wear life of the U1.5C nanocomposite coating at normal loads of 12 and 15 N, respectively. It was observed that the U1.5C nanocomposite coating failed earlier just after ~4800 cycles at a normal load of 15 N, suggesting that the working load for the nanocomposite coating be 12 N at a linear speed of 0.1 m/s.

### 3.5. Development and Characterization of the Hybrid Nanocomposite Coating

From the above wear tests, it was concluded that an optimum loading of 1.5 wt.% of CNT’s in the UHMWPE polymer matrix resulted in improved tribological performance in terms of a better load-bearing capacity and wear resistance of the nanocomposite coating as compared to the pristine UHMWPE coating. Therefore, we proceeded further to develop a hybrid nanocomposite coating by adding the different loadings (0.5, 1.5, 3 and 5 wt.%) of our second nano-filler, namely, hydroxyapatite (HA), by keeping the CNT’s content constant at 1.5 wt.%. The developed hybrid nanocomposite coating is evaluated for its mechanical/tribological properties and biocompatibility as discussed below.

#### 3.5.1. Dispersion Analysis of HA in U1.5C Matrix Using SEM

**Figure 7:**
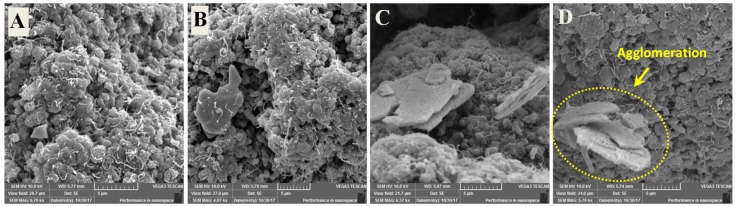
SEM images showing the dispersion of (**A**) 0.5 wt.%, (**B**) 1.5 wt.%, (**C**) 3 wt.% and (**D**) 5 wt.% of hydroxyapatite (HA) in UHMWPE/1.5 wt.% CNT’s matrix.

Figure 7 below shows the SEM images of the U1.5C powders reinforced with the different loadings (0.5, 1.5, 3, and 5 wt.%) of HA. It can be observed from Figure 7A–C that the HA platelets have been disentangled properly resulting in their uniform dispersion without any agglomerates. However, as the loading of HA was increased to 5 wt.%, a few agglomerates of HA were observed as can be seen in Figure 7D.

#### 3.5.2. Tribological Characterization of UHMWPE Hybrid Nanocomposite Coating

**Figure 8:**
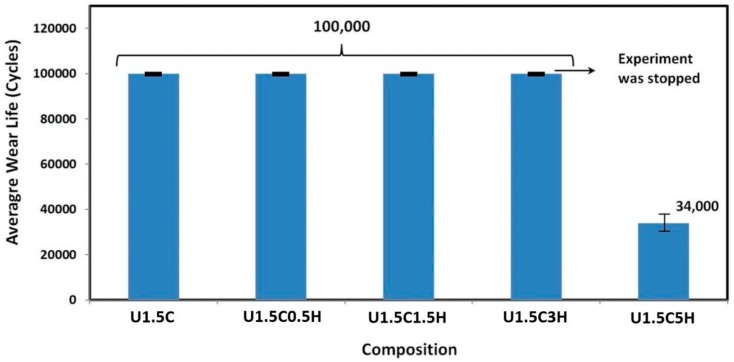
Average wear life of hybrid nanocomposite coatings, recorded after a wear test conducted at a normal load of 12 N, and a sliding velocity of 0.1 m/s for 100,000 cycles.

Different loadings of hydroxyapatite (0.5, 1.5, 3, 5 wt.%) were integrated into the U1.5C matrix to improve the bioactivity and osteoconductivity of the coating. In addition, studies have also shown an improvement in mechanical properties upon the addition of HA in the UHMWPE matrix [29]. Hence, to investigate the mechanical/tribological properties of the developed hybrid nanocomposite coating, it was deposited on pure titanium substrates following the coating procedure explained in the experimental section and initially, wear tests were conducted for 100,000 cycles at a normal load of 12 N with a sliding velocity of 0.1 m/s. It can be seen from Figure 8, that hybrid nanocomposite coatings of U1.5C reinforced with 0.5, 1.5, and 3 wt.% HA, completed the 100,000 cycles test without failure. However, increasing the percentage of HA to 5 wt.% resulted in the failure of the coating after ~34,000 cycles. The failure was confirmed from the EDX spectrum on the wear track where a peak of Ti was clearly seen, suggesting a metal to metal contact. This failure can be attributed to the agglomerations and the pile-up of HA plates as seen in the SEM image (Figure 7D). These agglomerations of the HA may result in the non-uniform properties throughout the polymer matrix resulting in a severe material pull-out leading to high wear.

Since the hybrid nanocomposite coating of U1.5C reinforced with 0.5, 1.5, and 3 wt.% of HA did not fail even at 100,000 cycles and to ascertain the wear resistance of these hybrid nanocomposite coating for prolonged durations, the above-mentioned composition coatings were further tested for a prolonged period of 250,000 cycles, at a load of 12 N and a sliding velocity of 0.1 m/s.

**Figure 9:**
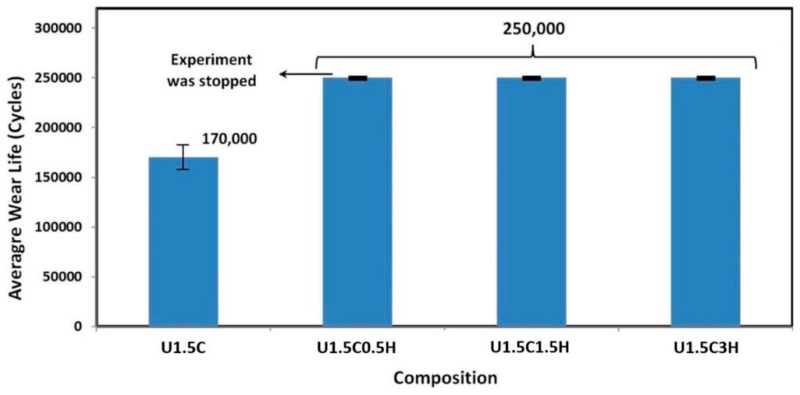
Average wear life of hybrid nanocomposite coatings, recorded after a wear test conducted at a normal load of 12 N, and a sliding velocity of 0.1 m/s for 250,000 cycles.

**Figure 10:**
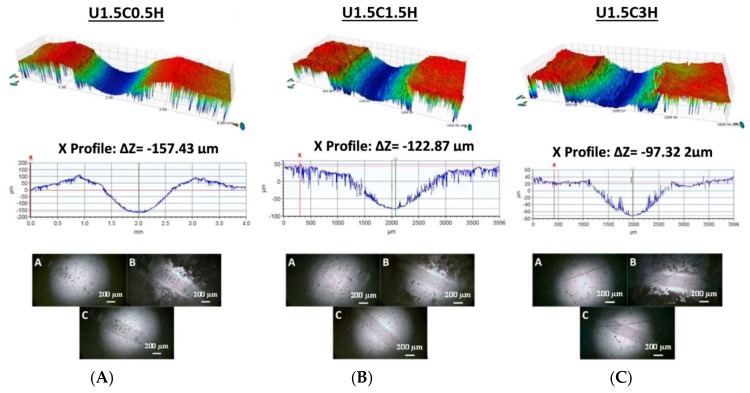
3D and 2D profiles and counterface ball images after the wear test conducted on UHMWPE hybrid nanocomposite coating, recorded at a normal load of 12 N and a sliding velocity of 0.1 m/s for 250,000 cycles. (**A**) U1.5C0.5H; (**B**) U1.5C1.5H; (**C**) U1.5C3H.

It can be seen from Figure 9, the U1.5C nanocomposite coating failed earlier, presenting an average wear life of ~170,000 cycles. However, all the tested hybrid nanocomposite coatings with 0.5, 1.5, and 3 wt.% of HA completed the 250,000 cycles test without failure. The 3D, 2D profiles and counterface ball images of a typical test are shown in Figure 10.

**Figure 11:**
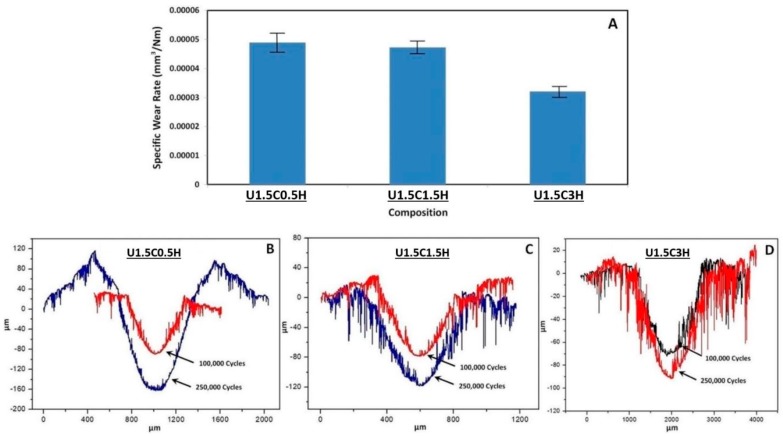
(**A**) Specific wear rates and 2D profiles of hybrid nanocomposite coatings recorded at a tribological test conducted for 100,000 cycles and 250,000 cycles to evaluate the difference in track depth. (**B**) U1.5C0.5H; (**C**) U1.5C1.5H; (**D**) U1.5C3H.

Since the U1.5C hybrid nanocomposite coating reinforced with different loadings (0.5, 1.5, and 3 wt.%) of HA did not fail after a wear test conducted for 100,000 cycles and also after a wear test conducted for 250,000 cycles, we evaluated the change in the wear depth with an increase in the number of cycles for each of the hybrid nanocomposite coating to ascertain their individual wear resistances. Figure 11B–D shows the comparison of the 2D wear track profiles for all three compositions after a wear test of 100,000 cycles and 250,000 cycles, respectively. 

It was observed that with a rise in the HA content from 0.5 to 3 wt.%, the wear depth decreased from 75.7% to 39.2% when the number of cycles was increased from 100,000 cycles to 250,000 cycles for 0.5 wt.% HA and 3wt% HA, respectively. Furthermore, a 70.7% and 42% increase in wear volume is seen for 0.5 wt.% HA and 3% HA upon increasing the number of cycles from 100,000 cycles to 250,000 cycles. Specific wear rates were calculated as shown in Figure 11A it was observed that 3 wt.% HA had the lowest specific wear rate, signifying the 3 wt.% HA as a more wear-resistant coating compared to 0.5 wt.% and 1.5 wt.%.

#### 3.5.3. Tribological Characterization of the Optimized Hybrid Nanocomposite Coating Deposited on Titanium Alloys

**Figure 12:**
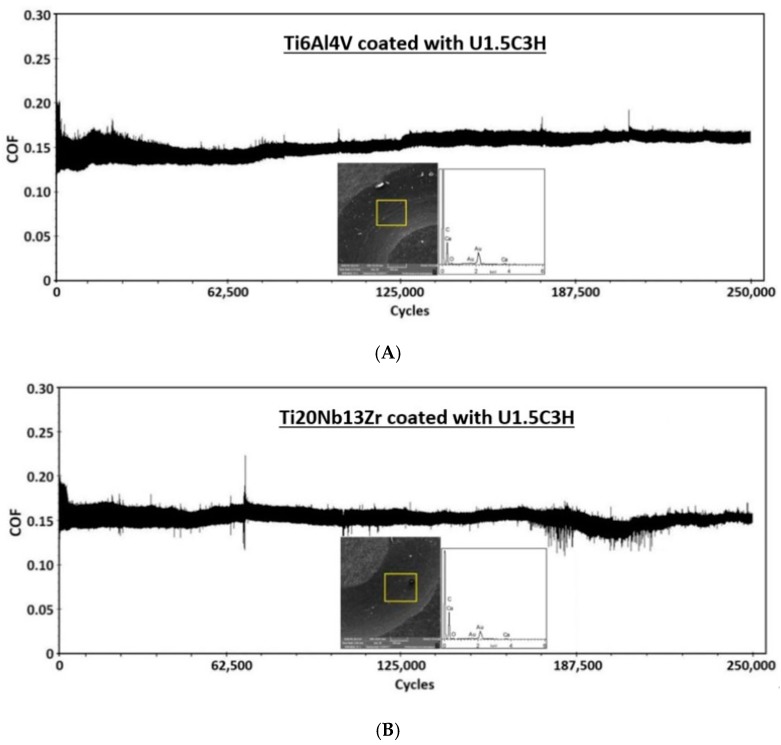
Typical COF graph, SEM images of wear track and EDX analysis of hybrid nanocomposite coating deposited on (**A**) Ti6Al4V (**B**) Ti20Nb13Zr, recorded after a wear test performed at a normal load of 12N and a sliding velocity of 0.1 m/s for 250,000 cycles.

The optimized hybrid nanocomposite coating (U1.5C3H) was further deposited on titanium alloys (Ti6Al4V and Ti20Nb13Zr) to study the effect of substrate on the tribological performance of the hybrid coating. Three samples of each titanium alloy were coated with UHMWPE reinforced with 1.5 wt.% CNT’s and 3 wt.% HA and tribologically characterized using the same test parameters for a normal loading condition and sliding velocity of 12 N and 0.1 m/s, respectively. No certain substrate effect was observed. The coating completed the 250,000 cycles test without failure for all the three tests for both titanium alloys. Figure 12 shows the COF graph along with the wear track and EDX analysis for one of the tested samples on different titanium alloys.

### 3.6. Hardness Evaluation of the Hybrid Nanocomposite Coatings

**Figure 13:**
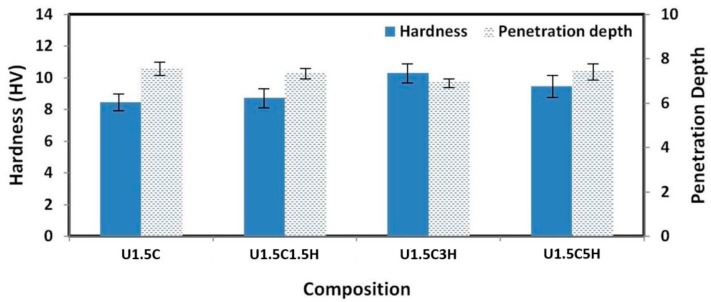
Vickers hardness and penetration depth vs. composition for nanocomposite and hybrid nanocomposite coatings.

Figure 13 shows the change in hardness and penetration depth for different compositions of HA added to UHMWPE reinforced with 1.5 wt.% CNT’s. A 22.1% increase in the hardness value and a 9% decrease in penetration depth is observed for 0 wt.% HA to 3 wt.% HA. The hardness and penetration depth was calculated from the average of 20 different indentations made at different locations on the sample. The improved tribological performance for 3 wt.% HA can be attributed to the increase in hardness. A drop in the hardness value and an increase in penetration depth was observed for 5 wt.% HA this could be due to agglomerations of HA plates, as seen during SEM analysis.

### 3.7. Evaluation of Scratch Resistance for Pristine UHMWPE, UHMWPE Reinforced with 1.5 wt.% of CNT’s and UHMWPE Reinforced with 1.5 wt.% CNT’s and 3 wt.% HA Coatings

In order to obtain a better understanding of the optimized coating, linear scratch tests were conducted on pristine UHMWPE, UHMWPE reinforced with 1.5 wt.% CNT’s nanocomposite coating and UHMWPE reinforced with 1.5 wt.% CNT’s and 3 wt.% HA hybrid nanocomposite coatings.

**Figure 14:**
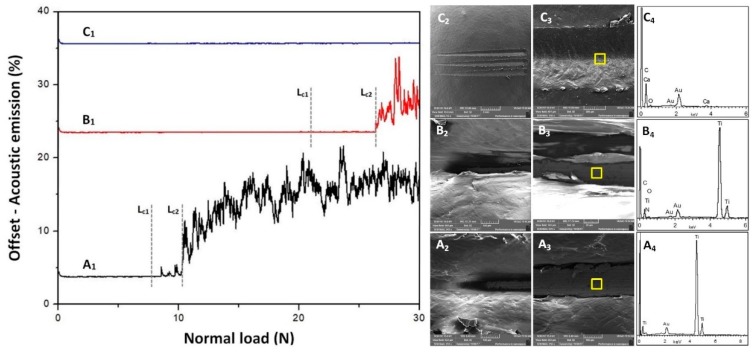
Acoustic emission with respect to the applied normal load from 0 to 30 N and SEM image of scratch with EDX analysis for (A_1_/A_2_/A_3_/A_4_) Pristine UHMWPE (B_1_/B_2_/B_3_/B_4_) U1.5C (C_1_/C_2_/C_3_/C_4_) U1.5C3H.

Figure 14A_1_ shows the acoustic emission with respect to the applied normal load for pristine UHMWPE coated on pure titanium. L_c1_ and L_c2_ represent the initial load of failure and a complete load of failure, respectively. At an average load of ~7.3 N initial failure occurs, and the diamond tip penetrates the coating as seen in the SEM image in Figure 14A_2_. Complete failure of the coating occurs at a load of ~10.2 N considerable peeling off, and plowing is observed, and clear signs of plastic deformation were produced along the edges of the scratch, as shown in the SEM images in Figure 14A_3_ and EDX analysis Figure 14A_4_ confirms the exposure of titanium.

The nanocomposite coating of UHMWPE reinforced with 1.5 wt.% CNT’s initially failed at an average normal load of 21.8 N and reaches complete failure at an average normal load of 26.4 N (Figure 14B_1_), which shows a significant increase in the scratch resistance of the nanocomposite coating, as compared to pristine UHMWPE, suggesting that the addition of CNT’s tremendously improves the adhesive strength/scratch resistance of the coating. Figure 14B_2_–B_4_) shows the SEM image along with the EDX analysis at the location of the failure.

The hybrid nanocomposite coating of UHMWPE reinforced with 1.5 wt.% CNT’s and 3 wt.% HA, did not fail during a linear progressive scratch test, as shown in Figure 14C_1_. Acoustic emission remains constant throughout the test indicating the indenter not being able to penetrate the coating until a normal load of 30 N, implying a very adhesive and a scratch-resistant coating. Figure 14C_2_ shows the full length of the scratch, and Figure 14C_3_ shows a location of the scratch at ~29 N normal load.

### 3.8. Comparison of the Tribological Performance of the Bare Substrates with the Substrates Coated with the Developed Optimized Hybrid Nanocomposite Coating

**Figure 15:**
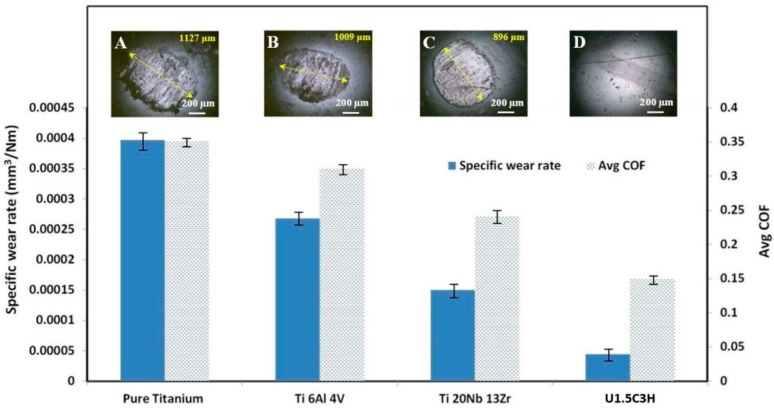
Specific wear rates/coefficient of friction for bare substrates and the hybrid nanocomposite coating tested under at a normal load of 12 N and a sliding velocity of 0.1 m/s. (**A**) Pure Titanium; (**B**) Ti6Al4V; (**C**) Ti20Nb13Zr; (**D**) U1.5C3H.

Tribological performance in terms of specific wear rate (SWR) and coefficient of friction (COF) of the developed optimized hybrid nanocomposite coating was compared to that of the different bare substrates used in this study to highlight the efficiency of the developed coatings in improving the wear life of the substrates and its capability to protect the substrates from wear and tear. Specific wear rates (SWR) were calculated for the bare substrates, and the optimized UHMWPE hybrid nanocomposite coating reinforced with 1.5 wt.% CNT’s and 3 wt.% HA. The coating had a considerable low SWR and low COF as compared to the bare substrates, as shown in Figure 15.

It is to be noted that in a tribological application, the wear life of both the mating surfaces is of utmost importance. The coating or surface modification done to improve the tribological properties of one of the mating surfaces should be able to protect even the counterface material from wear and tear for a successful tribological performance of the complete system. As can be seen from Figure 15, the specific wear rate and the COF for the developed hybrid nanocomposite coating are significantly lower as compared to those of the bare substrates. However, it is interesting to note that even the counterface ball which slid against the developed hybrid nanocomposite coating shows no signs of wear and tear, as can be clearly seen from the optical image of the ball (Figure 15D) recorded after a test of 250,000 cycles signifying the improved tribological performance of the coating to protect the complete system. However, the counterface balls sliding against the bare substrates show big scar marks only after a test run for 2400 cycles as shown in Figure 15A–C. Therefore, the developed and optimized hybrid nanocomposite coating was not only successful in reducing the COF and SWR of the titanium alloys but also was effective in protecting the wear and tear of the counterface.

### 3.9. Corrosion Test of Bare Substrate, Substrates with Nanocomposite and the Hybrid Nanocomposite Coatings

**Figure 16:**
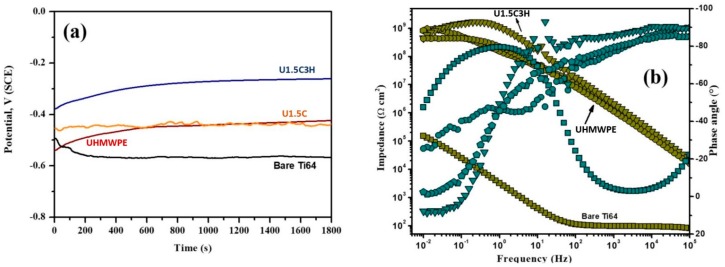
(**a**) Monitoring of open circuit potential values. (**b**) Electrochemical impedance spectroscopy.

Figure 16a shows the monitoring of open circuit potential (OCP) values for uncoated and coated Ti6aAl4V alloy samples in the SBF medium. Primarily, the uncoated Ti6Al4V sample consumes ∼400 s to reach about −542 mV vs. SCE, then the OCP value becomes steadily stable after a definite period, representing the stabilization of the passive layer. Besides, OCP of coated samples increases more quickly with the time at the initial 500 s, demonstrating compact coating, and then maintains a relatively higher steady value. It can be observed that the OCP values of Ti6Al4V with the 1.5 wt.% CNT’s and the hybrid nanocomposite incorporated UHMWPE coatings shifted to the noble direction and in particular, for the hybrid nanocomposite coating of UHMWPE reinforced with 1.5 wt.% CNT’s and 3 wt.% HA offered the noblest shift in potential. This behavior indicated that the corrosion protection performance of UHMWPE coatings is enhanced with the incorporation of 1.5 wt.% CNT’s and 3 wt.% HA. Further, to get a clear insight, EIS measurements are performed to validate the obtained OCP results.

Figure 16b represents the electrochemical impedance spectroscopic (EIS) data in Bode formats. Initial reflection of the Bode graphs of all of the coated samples suggests different EIS curves with the uncoated Ti6Al4V. For uncoated substrates, the large phase angle in mid- and low-frequency regions continued about −80°, while the slope of resistant curves was found to be about −1, representing the distinctive result of the capacitive behavior of the passive native layer. In contrast, for the case of coated Ti6Al4V samples, two-phase angle maxima were obtained in high and low-frequency areas, which indicates the interaction of at least two-time constants associated with the two-layer structure of the coated Ti6Al4V samples. In particular, the coatings with the incorporation of 1.5 wt.% CNT’s and 3 wt.% HA exhibited the highest impedance values, which confirmed the enhanced behavior of the coatings. In general, in Bode plots, a higher impedance modulus (Z) at a lower frequency region indicates a higher corrosion resistance of a metal substrate [30]. The impedance in the low-frequency region for the coated Ti6Al4V substrates appears to be nearly four orders of magnitude higher than that shown by the uncoated Ti6Al4V substrate. The higher impedance is possibly due to a barrier performance where the coating is an obstructive admittance of the hostile electrolytic species toward the metal/coating interface. The quantitative analysis of EIS data needs fitting with an accurate equivalent circuit model. Thus, an equivalent circuit with two-time constants was utilized to analyze the obtained EIS curves of the coated specimens [31]. The fitted equivalent circuit model denoted as R_s_ (R_ct_Q_1_) (R_f_Q_2_) contains two combinations of resistors and capacitors with the solution resistance. Here, *R_s_* signifies the solution resistance, which relates to the ohmic resistance of the system, *R_ct_*, and *R_f_* represent the charge transfer resistance and film resistance. *Q*_1_ and *Q*_2_ represent the capacitance of the double layer and the film, respectively. Instead of a pure capacitance, a constant phase element (CPE) was used as an ideal capacitive behavior in real solutions is not observed. Furthermore, its use minimizes error and provides more detailed information about the coating’s non-ideal dielectric properties. The CPE in the current work was calculated using the following equation [31]:

Z = Y_0_^−1^ (jω)^−n^ (2)

where *ω* is the angular frequency (2πf), *Y* is a proportionality factor, and *n* is the deviation parameter that is associated with the surface roughness. Here, n = 1 for an ideal capacitor where Q_1_ = C_dl_. R_ct_ value of the Ti6Al4V substrates with coatings increased from 151.26 kΩ cm^2^ for bare to 422.35, 853.25, and 914.54 MΩ cm^2^ for the UHMWPE, U1.5C and U1.5C3H nanocomposite, respectively, representing improved and noteworthy anticorrosion behavior. Generally, R_f_ values could be influenced by the number of pores/capillary networks in the coatings surface, through which the hostile species from the solution spread the metal/coating interface. The highest R_f_ value of 1.18 GΩ cm^2^ was obtained for the Ti6Al4V coated with U1.5C3H, indicating that this is the least porous coating. Hence, the inclusion of nanocomposite in the UHMWPE matrix appears to pointedly reduce the porosity of the UHMWPE coatings by covering the micro-cracks and voids inside the coating. The high impedance values significantly delayed the dissemination of hostile species, and subsequently improved the surface protective performance of the coatings against corrosion. Further, the increase in *Q*_1_ and Q_2_ values is associated with the diffusion of active species at the interface and expands the delaminated area. A comparison of the *Q*_1_ and Q_2_ values of the coated Ti6Al4V indicated that nanocomposite samples had the lowest *Q*_1_ and Q_2_ values (1.5 and 8.6 × 10^−3^ µF cm^−2^), which inferred that this coating retained a stable coating/metal interface devoid of any corrosion. Based on the electrochemical results, it can be revealed that the hybrid UHMWPE nanocomposite coatings exhibit better corrosion protection performance than pure UHMWPE and uncoated Ti6Al4V sample in SBF medium.

## 4. Conclusions

In this study, a polymer-based hybrid nanocomposite coating was developed by reinforcing UHMWPE with different loadings of carbon nanotubes (CNT’s) (0.5, 1.5, and 3 wt.%) to enhance the tribological performance of the polymer coating and hydroxyapatite (HA) (0.5, 1.5, 3, and 5 wt.%) to improve the biocompatibility of the polymer coating. Pure titanium (Grade 2) and titanium alloys (Ti6Al4V and T20Nb13Zr) were used as substrates for the polymer coating. 

Tribological performance of pristine UHMWPE, UHMWPE/CNT’s nanocomposite coating and UHMWPE/CNT’s/HA hybrid nanocomposite coatings were evaluated by using a ball on disk configuration under dry conditions and room temperature. The following conclusions can be deduced from these experimental results:

1. Pristine UHMWPE coating failed at a normal load of 12 N and a sliding velocity of 0.1 m/s, showing a wear life of ~3600 cycles.
2. UHMWPE reinforced with 1.5 wt.% CNT’s did not fail the 50,000 cycles wear test, whereas 0.5 wt.% CNT’s and 3 wt.% CNT’s failed at 28,000 cycles and 7000 cycles, respectively, for a normal load of 12 N and sliding velocity of 0.1 m/s.
3. Among the three combinations of the developed Hybrid nanocomposite coatings, for a 250,000 cycles wear test and a normal load of 12 N and sliding velocity of 0.1 m/s, UHMWPE reinforced with 1.5 wt.% CNT’s and 3 wt.% HA exhibited excellent tribological performance in terms of lower track depth and protecting the wear and tear of the counterface ball.
4. Corrosion test for the optimized hybrid nanocomposite coating revealed a noble shift in potential for the OCP value and the highest impedance value, which confirms the enhanced surface protective performance of the coating against corrosion in SBF medium.

## Figures and Tables

**Table 1:** Codes assigned to each of the compositions prepared in the present study.

Composition	Code
Pristine UHMWPE	UHMWPE
UHMWPE/0.5 wt.% CNT’s	U0.5C
UHMWPE/1.5 wt.% CNT’s	U1.5C
UHMWPE/3 wt.% CNT’s	U3C
UHMWPE/1.5 wt.% CNT’s/0.5 wt.% HA	U1.5C0.5H
UHMWPE/1.5 wt.% CNT’s/1.5 wt.% HA	U1.5C1.5H
UHMWPE/1.5 wt.% CNT’s/3 wt.% HA	U1.5C3H
UHMWPE/1.5 wt.% CNT’s/5 wt.% HA	U1.5C5H

## References

[1] Uwais Z.A., Hussein M.A., Samad M.A., Al-Aqeeli N. (2017). Surface Modification of Metallic B iomaterials for Better Tribological Properties: A Review. Arab. J. Sci. Eng..

[2] Luo Y., Ge S., Jin Z., Fisher J. (2009). Effect of surface modification on surface properties and tribological behaviours of titanium alloys. Proc. Inst. Mech. Eng. Part J.

[3] Minn M., Sinha S.K. (2008). DLC and UHMWPE as hard/soft composite film on Si for improved tribological performance. Surf. Coatings Technol..

[4] Bakshi S.R., Balani K., Laha T., Tercero J., Agarwal A. (2007). The Nanomechanical and Nanoscratch Properties of MWNT-Reinforced Ultrahigh-Molecular-Weight Polyethylene Coatings. JOM.

[5] Ali A.B., Samad M.A., Merah N. (2017). UHMWPE Hybrid Nanocomposites for Improved Tribological Performance Under Dry and Water-Lubricated Sliding Conditions. Tribol. Lett..

[6] Kurtz S.M., Oral E. (2016). In Vivo Oxidation of UHMWPE. UHMWPE Biomaterials Handbook.

[7] Suhendra N., Stachowiak G.W. (2007). Computational model of asperity contact for the prediction of UHMWPE mechanical and wear behaviour in total hip joint replacements. Tribol. Lett..

[8] Samad M.A., Sinha S.K. (2011). Dry sliding and boundary lubrication performance of a UHMWPE/CNTs nanocomposite coating on steel substrates at elevated temperatures. Wear.

[9] Baena J., Wu J., Peng Z. (2015). Wear Performance of UHMWPE and Reinforced UHMWPE Composites in Arthroplasty Applications: A Review. Lubricants.

[10] Panjwani B., Satyanarayana N., Sinha S.K. (2011). Tribological characterization of a biocompatible thin film of UHMWPE on Ti6Al4V and the effects of PFPE as top lubricating layer. J. Mech. Behav. Biomed. Mater..

[11] Zhang L., Wang Q., Liu G., Guo W., Ye B., Li W., Jiang H., Ding W. (2018). Tribological Behavior of Carbon Nanotube-Reinforced AZ91D Composites Processed by Cyclic Extrusion and Compression. Tribol. Lett..

[12] Tai Z., Chen Y., An Y., Yan X., Xue Q. (2012). Tribological behavior of UHMWPE reinforced with graphene oxide nanosheets. Tribol. Lett..

[13] Lahiri D., Dua R., Zhang C., de Socarraz-Novoa I., Bhat A., Ramaswamy S., Agarwal A. (2012). Graphene Nanoplatelet-Induced Strengthening of UltraHigh Molecular Weight Polyethylene and Biocompatibility In vitro. ACS Appl. Mater. Interfaces.

[14] Plumlee K., Schwartz C.J. (2009). Improved wear resistance of orthopaedic UHMWPE by reinforcement with zirconium particles. Wear.

[15] Bakshi S.R., Balani K., Laha T., Tercero J., Agarwal A., Anderson R., Laha T., Andara M., Tercero J., Crumpler E. (2007). Plasma-sprayed carbon nanotube reinforced hydroxyapatite coatings and their interaction with human osteoblasts in vitro. Biomaterials.

[16] Sreekanth D., Rameshbabu N. (2012). Development and characterization of MgO/hydroxyapatite composite coating on AZ31 magnesium alloy by plasma electrolytic oxidation coupled with electrophoretic deposition. Mater. Lett..

[17] Lu Y.-P., Li M.-S., Li S.-T., Wang Z.-G., Zhu R.-F. (2004). Plasma-sprayed hydroxyapatite+titania composite bond coat for hydroxyapatite coating on titanium substrate. Biomaterials.

[18] Li Y., Li Q., Zhu S., Luo E., Li J., Feng G., Liao Y., Hu J. (2010). The effect of strontium-substituted hydroxyapatite coating on implant fixation in ovariectomized rats. Biomaterials.

[19] Kim H.-W., Knowles J.C., Kim H.-E. (2004). Hydroxyapatite/poly(ε-caprolactone) composite coatings on hydroxyapatite porous bone scaffold for drug delivery. Biomaterials.

[20] Chen X., Zhang B., Gong Y., Zhou P., Li H. (2018). Mechanical properties of nanodiamond-reinforced hydroxyapatite composite coatings deposited by suspension plasma spraying. Appl. Surf. Sci..

[21] Balani K., Chen Y., Harimkar S.P., Dahotre N.B., Agarwal A. (2007). Tribological behavior of plasma-sprayed carbon nanotube-reinforced hydroxyapatite coating in physiological solution. Acta Biomater..

[22] Hussein M.A., Suryanarayana C., Al-Aqeeli N. (2015). Fabrication of nano-grained Ti-Nb-Zr biomaterials using spark plasma sintering. Mater. Des..

[23] Hussein M., Kumar M., Drew R., Al-Aqeeli N. (2017). Electrochemical Corrosion and In Vitro Bioactivity of Nano-Grained Biomedical Ti-20Nb-13Zr Alloy in a Simulated Body Fluid. Materials.

[24] Paz A., Guadarrama D., López M., E González J., Brizuela N., Aragón J. (2012). A comparative study of hydroxyapatite nanoparticles synthesized by different routes. Química Nova.

[25] Kwok C.T., Wong P.K., Cheng F.T., Man H.C. (2009). Characterization and corrosion behavior of hydroxyapatite coatings on Ti6Al4V fabricated by electrophoretic deposition. Appl. Surf. Sci..

[26] Azam M.U., Samad M.A. (2018). A novel organoclay reinforced UHMWPE nanocomposite coating for tribological applications. Prog. Org. Coat..

[27] Wang Y., Vasileva D., Zustiak S.P., Kuljanishvili I. (2017). Raman spectroscopy enabled investigation of carbon nanotubes quality upon dispersion in aqueous environments. Biointerphases.

[28] Halley P., Murphy M., Martin D., Mcnally T., Po P., Bell S.E.J., Brennan G.P., Bein D., Lemoine P., Paul J. (2005). Polyethylene multiwalled carbon nanotube composites. Polymer.

[29] Fang L., Leng Y., Gao P. (2006). Processing and mechanical properties of HA/UHMWPE nanocomposites. Biomaterials.

[30] Williams G., McMurray H.N. (2012). Inhibition of filiform corrosion on organic-coated AA2024-T3 by smart-release cation and anion-exchange pigments. Electrochim. Acta.

[31] Yuan X., Yue Z.F., Chen X., Wen S.F., Li L., Feng T. (2015). EIS study of effective capacitance and water uptake behaviors of silicone-epoxy hybrid coatings on mild steel. Prog. Org. Coat..

